# Occipital artery–posterior inferior cerebellar artery bypass for anterior inferior cerebellar artery stenosis with repeated cerebellar ischemic stroke: a case report

**DOI:** 10.1186/s13256-025-05092-7

**Published:** 2025-02-18

**Authors:** Hiroki Eguchi, Takashi Arai, Takakazu Kawamata

**Affiliations:** 1 Department of Neurosurgery, Tokyo Women’s Medical University Hospital, 8-1 Kawada-Cho, Shinjuku-Ku, Tokyo, Japan; 2 Department of Neurosurgery, Makita General Hospital, Tokyo, Japan

**Keywords:** Cerebellar infarction, Anastomosis, OA-PICA bypass

## Abstract

**Background:**

Vertebrobasilar insufficiency revascularization has long been a preventive treatment for cerebral infarction. However, no studies have demonstrated the efficacy of revascularization in patients with cerebellar ischemia.

**Case presentation:**

We present the case of a 77-year-old Japanese man who experienced seven recurrent cerebellar infarctions over 2 years. Severe stenosis was noted at the origin of the common trunk of the posterior inferior cerebellar artery and anterior inferior cerebellar artery. Patients with cerebral infarctions are resistant to medical treatment. Blood flow evaluation showed that the area of reduced cerebrovascular reactivity corresponded to the area where repeated small infarctions occurred. An occipital artery–posterior inferior cerebellar artery bypass was performed to prevent another infarction. Postoperative single-photon emission computed tomography showed an improved cerebrovascular reactivity. No ischemic events occurred during 2 years of a postoperative follow-up period.

**Conclusion:**

Occipital artery–posterior inferior cerebellar artery bypass is an effective treatment method for vascular stenosis and decreased blood circulation due to posterior circulation ischemia.

## Background

Hemodynamic failure, a major factor in vertebrobasilar insufficiency (VBI) [1], is often caused by organic factors such as stenosis of the vertebral or basilar arteries. Endovascular treatments, such as percutaneous transluminal angioplasty and stent placement or superficial temporal artery (STA)–superior cerebellar artery (SCA) bypass, are treatment options for VBI. Occipital artery (OA)–posterior inferior cerebellar artery (PICA) bypass has been performed to improve blood circulation in cases of VBI since its utility was first reported by Ausman et al. [2]. However, the frequency of OA-PICA bypass has decreased in recent years owing to technical difficulties, few indications such as safe bypass for aneurysm treatment, and the widespread use of endovascular treatment [2].

In this study, we describe a case of repeated cerebellar infarction due to common-origin anterior inferior cerebellar artery (AICA)–PICA stenosis. As cerebral blood flow testing using single-photon emission computed tomography (SPECT) demonstrated significantly decreased cerebrovascular reactivity (CVR), we performed an OA-PICA bypass, which yielded good results. We also review the related literature.

## Case presentation

**Fig. 1:**
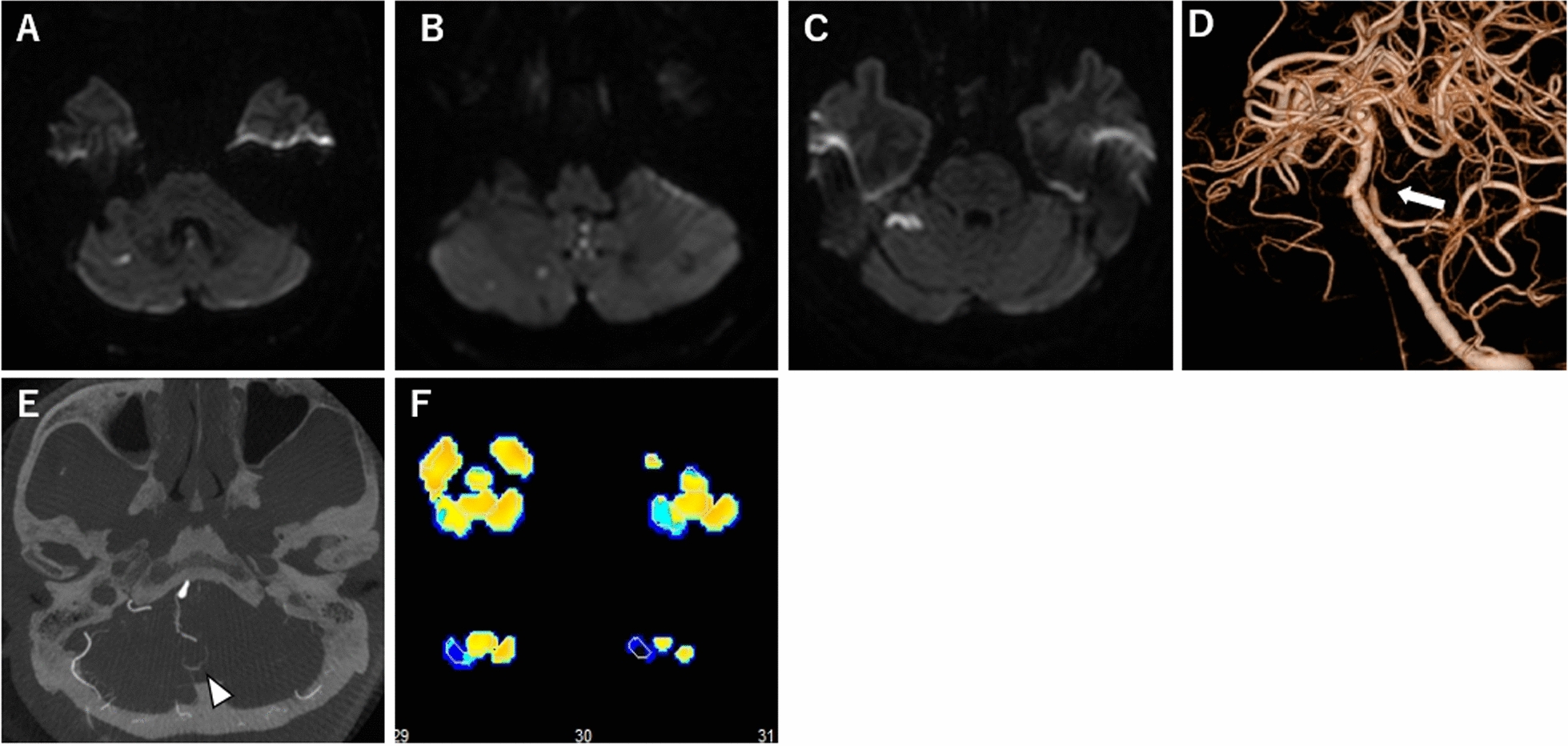
Patient’s preoperative course. **A** Diffusion-weighted image showing right cerebellar hemisphere infarction at disease onset. **B** Diffusion-weighted image showing the recurrence of right cerebellar hemisphere infarction at 2 years after disease onset. **C** Diffusion-weighted image showing relapse 1 month after recurrence. **D** Three-dimensional angiogram showing severe stenosis at the origin of the right anterior inferior cerebellar artery–posterior inferior cerebellar artery common trunk. **E** Identification of the peripheral location of the anterior inferior cerebellar artery–posterior inferior cerebellar artery (white arrowhead). **F** Decreased cerebrovascular reactivity in the right cerebellar hemisphere

Our patient was a 77-year-old Japanese man with a history of hypertension and diabetes mellitus. He developed a right cerebellar hemisphere infarction and presented with mild lightheadedness and vomiting. Magnetic resonance angiography revealed mild stenosis of the V4 portion of the right vertebral artery. Cilostazol 200 mg was initiated; however, 4 months later, the patient experienced two transient ischemic attack (TIA) episodes, with the chief complaint of lightheadedness. Aspirin 100 mg was added, but a right cerebellar infarction recurred 1 year after the initial diagnosis; therefore, it was switched to prasugrel. Subsequently, he developed a right cerebellar hemisphere infarction four times within 6 months and was repeatedly hospitalized. Cerebral angiography revealed a variation in the right AICA-PICA common trunk with severe stenosis at its origin. SPECT revealed an area of misery perfusion with a CVR of less than 10% (and blood steal phenomenon of less than 0%) in the right cerebellar hemisphere, which was the site of repeated cerebral infarctions (Fig. 1).

On the basis of these findings, we determined that revascularization would prevent the recurrence of cerebral infarction, and we decided to perform OA-PICA bypass surgery.

**Fig. 2:**
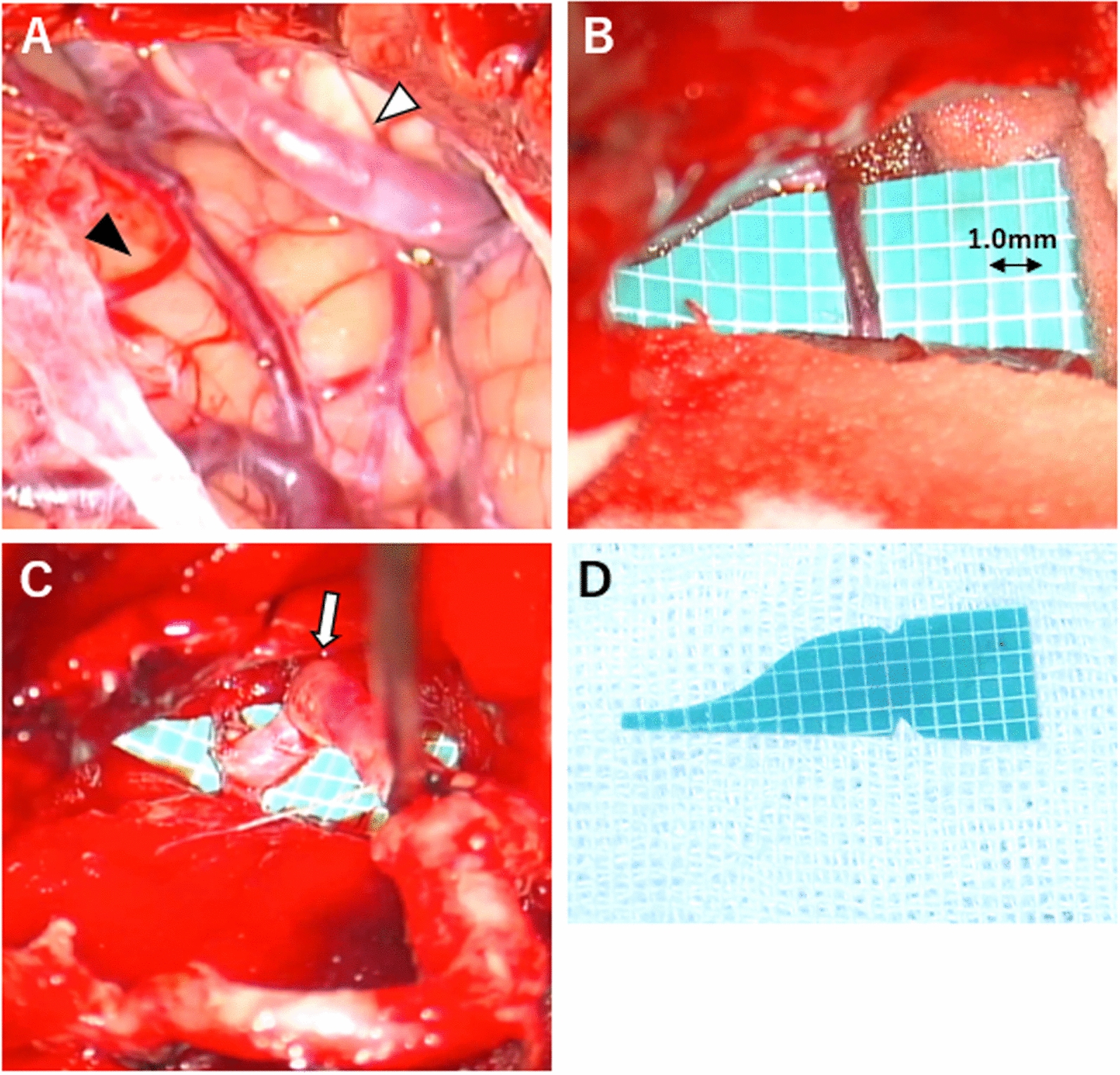
Occipital artery–posterior inferior cerebellar artery bypass. **A** The dura was incised and the target blood vessels were identified, including the target posterior inferior cerebellar artery (black arrowhead) and contralateral posterior inferior cerebellar artery (white arrowhead). **B** Recipient measuring 0.8 mm and rubber sheet with a 1.0-mm-squared grid. **C** Post-anastomotic occipital artery (white arrow). **D** Original rubber sheet

The patient was placed in the park-bench position, and a hockey stick-shaped incision was made from the second cervical vertebra to the inion and mastoid processes. The OA was anastomosed to the PICA (caudal segment), which was 0.8 mm long. Single interrupted 10–0 nylon sutures were placed, and patency was confirmed using indocyanine green angiography (isolation time, 35 min). No postoperative hyperperfusion or other complications were observed. The patient was discharged home on postoperative day 21, without any neurological symptoms (Fig. 2).

**Fig. 3:**
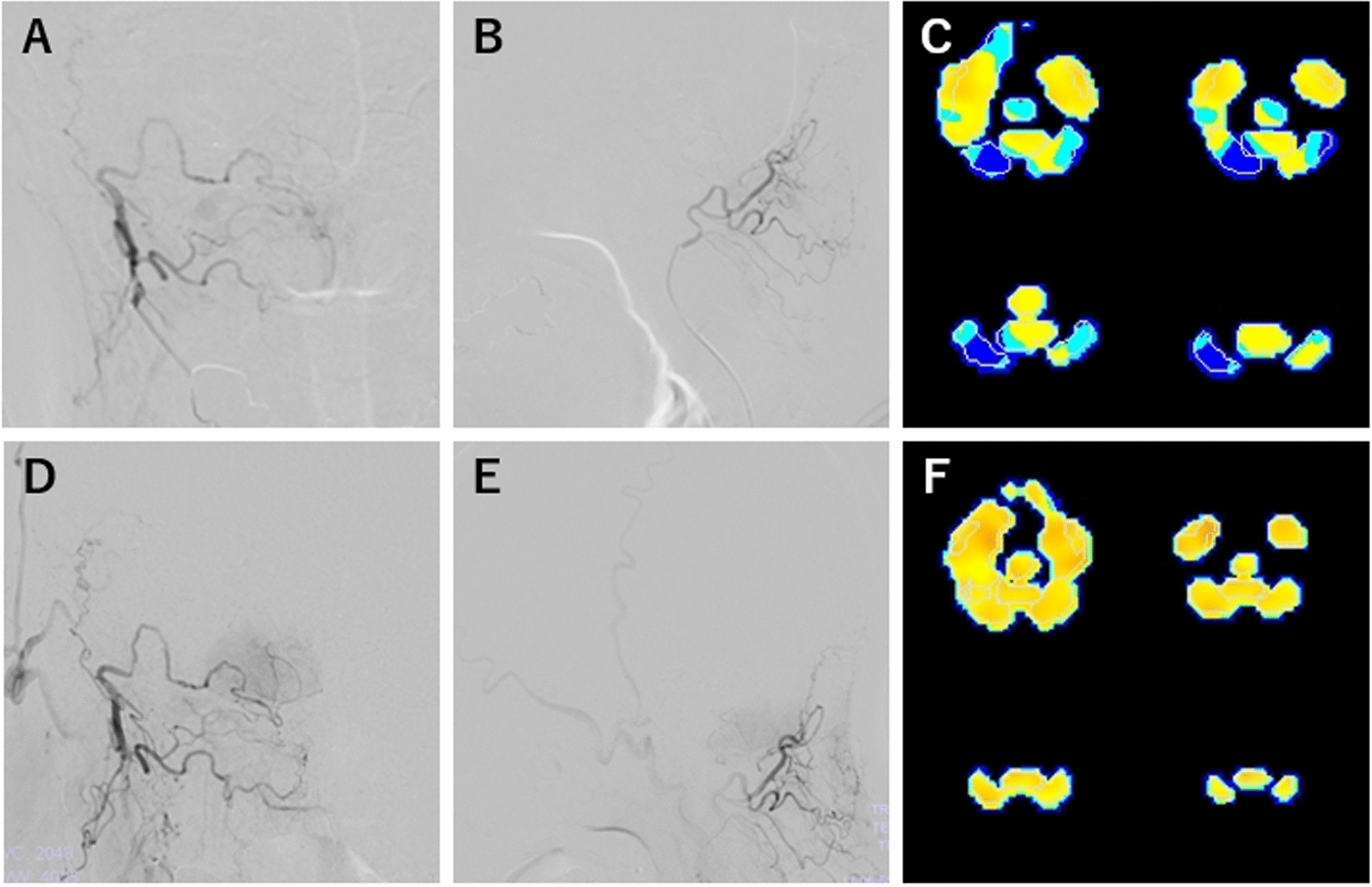
Patient’s postoperative course. **A**, **B** Angiography (frontal/lateral) performed 8 months later showing good blood flow from the occipital artery to the cerebellar hemispheres. **C** Cerebrovascular reactivity performed at 8 months post-operation showing decreased blood flow in the bilateral cerebellar hemispheres. **D**, **E** Angiography (frontal/lateral) performed 1.5 years post-operation showing improved blood flow from the occipital artery to the cerebellar hemisphere. **F** Cerebrovascular reactivity performed 1 year post-operation showing no difference in blood flow between the bilateral cerebellar hemispheres

Postoperative angiography revealed good perfusion of the bypass vessels in the right cerebellar hemisphere. Moreover, SPECT showed preoperative CVR of the right cerebellar hemisphere of 39.7% (18.14/45.70) on the contralateral side that increased to 50.3% (10.28/20.43) at 8 months post-operation, indicating slight improvement. A total of 18 months later, angiography confirmed growth of the bypass blood vessels, and SPECT showed complete disappearance of laterality in the CVR of the cerebellar hemispheres. Preoperatively, the patient suffered four cerebellar infarctions within only 6 months; for 2 years post-operation, he experienced no episodes of cerebral infarction or TIA (Fig. 3).

### Patient informed consent

The patient provided written informed consent for the publication of his case.

## Discussion and conclusion

### Observations

VBI is less common in the posterior than in anterior circulation; however, its pathology remains unclear. TIA due to VBI is very likely to progress to cerebral infarction [3, 4]. Cartlidge et al. reported that 25–35% of TIA episodes of the vertebrobasilar artery system progressed to a brainstem infarction within 5 years. TIA are reportedly unstable [3].

In the event of hemodynamic compromise, which is often involved in the etiology of VBI, revascularization is expected to improve the cerebral circulation and the posterior circulatory area metabolism [5, 6].

In 1975, Ausman *et al*. [2] demonstrated the effectiveness of revascularization of the OA-PICA in vertebral and basilar artery lesions. Since then, Khodadad and Olteanu-Nerbe *et al*. reported that OA-PICA bypass for VBI can reduce the risk of cerebral infarction and eliminate TIA by improving abnormalities in the cerebral circulation and metabolism [7, 8]. In contrast, Asuman *et al*. performed 15 OA-PICA bypasses for VBI and achieved a therapeutic efficacy of only 53%; Hopkins *et al*. demonstrated the effectiveness of OA-PICA bypass for vertebral artery lesions only [2, 9]. However, no study has demonstrated the ability of OA-PICA bypass revascularization to improve cerebellar ischemia caused by abnormal blood flow to the SCA, AICA, and PICA, which supply blood to the cerebellum. Kuroda *et al*. reported that accurately creating blood flow in blood-poor areas is important for the development of bypass vessels during revascularization of ischemic stroke [10].

The differences in the previous treatment results may be attributed to the lack of SPECT and the inability to accurately demonstrate hemodynamic failure. Confirming the consistency of the anastomotic vessels and the area of poor circulation is important when determining the suitability of revascularization using the bypass technique for VBI. Therefore, anatomical information obtained from preoperative SPECT and digital subtraction angiography (DSA) is necessary. Techniques for evaluating hemodynamics have improved in recent years. We performed SPECT and accurately confirmed decreased blood flow in the lateral cerebellar hemisphere. We used angiography to determine the perfusion area of the stenotic AICA-PICA and compared it with SPECT results to confirm that it corresponded to the area of reduced perfusion. In this case, bypass vessels developed 2 years postoperatively. SPECT revealed improved blood flow in the affected cerebellar hemisphere, indicating good patency of the bypass vessels in the ischemic area.

In this case, AICA origin stenosis and PICA variation caused frequent cerebellar infarctions. The AICA-PICA variant is a well-known mutation in which the AICA provides a blood supply to the AICA and PICA circulation. This variant has been reported in approximately 24% of routine vertebral angiographic examinations [11]. There have been no reports of AICA-PICA variations associated with an increased risk of cerebral infarction. However, the frequency is not low, and the range of perfusion in this variant is high, which may potentially aggravate ischemia-associated symptoms. Among the lesions responsible for cerebellar infarction, those involving the PICA are approximately half arteriosclerotic and half embolic, whereas those involving the AICA are mostly arteriosclerotic [12]. If a cerebellar infarction occurs repeatedly in the same region, vascular evaluation using DSA should be considered.

OA-PICA bypass is difficult and requires the use of complicated technologies because of the rarity of the surgery versus STA-MCA bypass surgery, the deep anastomosis site, and the large number of anatomical variations in the PICA. In recent years, the STA-SCA has become the mainstream revascularization procedure aimed at improving blood flow in VBI. We assumed the recipient’s position preoperatively and performed fusion with the bone using three-dimensional DSA. Perioperatively, a sponge (ITL Pharma Co.) was placed under the drainer to create a shallower surgical field. Threads and needles do not adhere easily to sponges. A graduated rubber sheet was inserted to create an appropriate OA stump. Triangular incisions were made at both ends of the rubber sheet and the recipient was inserted into the incisions to restrict blood vessel mobility and facilitate anastomosis. OA-PICA bypass can be accomplished using preoperative angiography and a specially designed armamentarium.

### Lessons

This case demonstrated that OA-PICA bypass can effectively prevent the recurrence of cerebral infarction in cases of stenosis of the blood vessels feeding the PICA region, reducing blood flow in the return region.

## Data Availability

Not applicable.

## Abbreviations

AICA: Anterior inferior cerebellar artery
CVR: Cerebrovascular reactivity
DSA: Digital subtraction angiography
DWI: Diffusion-weighted image
OA: Occipital artery
PICA: Posterior inferior cerebellar artery
SCA: Superior cerebellar artery
SPECT: Single-photon emission computed tomography
STA: Superficial temporal artery
TIA: Transient ischemic attack
VBI: Vertebrobasilar insufficiency
